# Milk protein concentrate supplementation improved appetite, metabolic parameters, adipocytokines, and body composition in dieting women with obesity: a randomized controlled trial

**DOI:** 10.1186/s40795-024-00879-1

**Published:** 2024-06-03

**Authors:** Mahsa Elahikhah, Fatemeh Haidari, Saman Khalesi, Hajieh Shahbazian, Majid Mohammadshahi, Vahideh Aghamohammadi

**Affiliations:** 1 Department of Nutrition, School of Paramedical Sciences, Ahvaz Jundishapur University of Medical Sciences, Ahvaz, Iran; 2 Nutrition and Metabolic Diseases Research Center, Ahvaz Jundishapur University of Medical Sciences, Ahvaz, Iran; 3 School of Health, Medical and Applied Sciences, Central Queensland University, Brisbane, Australia; 4 Diabetes Research Center, Health Research Institute, Ahvaz Jundishapur University of Medical Sciences, Ahvaz, Iran; 5 Department of Nutrition, Khalkhal University of Medical Sciences, Khalkhal, Iran

**Keywords:** Milk protein, Obesity, Weight loss, Adipocytokines, Appetite, Glycaemic indices

## Abstract

**Background:**

Dairy consumption is associated with many health benefits. However, to our knowledge, no clinical trials examined the effects of milk protein concentrate (MPC) on metabolic health in overweight and obese adults. This study investigated the effect of supplementation with MPC on glycaemic status, lipid profile, biomarkers of inflammation, and anthropometric measurements in women with obesity under a weight loss diet.

**Methods:**

This is a single-blind, open-labelled, parallel-group, randomized trial. Forty-four healthy women with obesity were randomized into a control (*n* = 22) or MPC (*n* = 22) group. Participants in the MPC group were supplemented with 30 g of MPC per day for 8 weeks. Both groups were on a calorie-restricted diet plan with 800 Kcal lower intakes than their needs. Blood samples, dietary intake, and body composition were assessed before and after the intervention.

**Results:**

MPC group had a significantly lower body mass index (*P* = 0.009), waist circumference (*P* = 0.013), fat mass (*P* = 0.021), appetite score (*P* = 0.002), fasting blood sugar (*P* < 0.001), insulin (*P* = 0.027), low-density lipoprotein cholesterol (*P* = 0.025), and leptin (*P* = 0.014) levels and higher high-density lipoprotein cholesterol (*P* = 0.001) and adiponectin (*P* = 0.032) compared to the control group after supplementation. Lean body mass, total cholesterol, and triglyceride did not differ significantly (*P* > 0.05).

**Conclusion:**

Daily intake of 30 g of MPC for 8 weeks may improve several anthropometric and metabolic markers in women with obesity under a hypocaloric diet.

## Background

Overweight and obesity remain the biggest health concern worldwide, affecting about two billion people [1]. About 27% of the population in Iran is also overweight and obese, with women being more predisposed to the condition [2]. The enlarged adipose tissue often ensues with the infiltration of macrophages, which put the body in a pro-inflammatory state by increasing the production of cytokines like leptin and reducing the synthesis of adiponectin. This hormonal dysregulation may lead to insulin resistance due to impaired cell signalling, which could further disrupt the lipid profile, leading to chronic diseases such as type 2 diabetes, cardiovascular diseases (CVD), and some types of cancers [3]. Weight loss is crucial for the management of the condition and to reduce the risk of chronic diseases [4]. A low-calorie diet is required for an effective weight loss. Also, some dietary constituents may be used as supplements in weight loss diets. Protein supplements may be beneficial in the management of weight loss due to their high diet-induced thermogenesis [5], ability to induce satiety (by stimulating the release of cholecystokinin), and prolonged satiation (by slowing gastric emptying) [6, 7]. However, proteins from different sources have diverse metabolic effects [8]. Dairy products are a major source of high-value protein. Dairy protein is made up of 2 major classes of proteins: casein (80%) and whey (20%). They are both complete proteins containing all essential amino acids, but they differ in the way in which they are digested and absorbed [9]. Literature suggests that higher consumption of dairy products is associated with a lower risk of obesity, metabolic-related disorders, and CVD [10–14]. These benefits were also more prominent when low-fat dairy was investigated [15]. Milk protein concentrate (MPC) is developed from pasteurized skim milk through diafiltration, ultrafiltration, and spray drying. MPC contains milk proteins (whey and casein) in the same ratio found in milk, while much of its fat, salt, and lactose are removed, making it an excellent ingredient for enriching foods and beverages [16]. Research has shown that milk proteins, especially whey protein, exert beneficial effects on glycaemic control by increasing insulin response and lowering blood glucose [17]. An animal study also reported a greater weight-reducing effect of MPC compared to that of whey or casein alone [18]. Milk intake has also been reported to reduce cholesterol levels and inflammation [19, 20]. However, to our knowledge, no clinical trial study has ever been carried out to examine the effects of MPC on metabolic health in overweight and obese adults. Therefore, in a single-blind, randomized clinical trial study we investigated the effect of a daily intake of 30 g of MPC for 8 weeks on glycaemic status, lipid profile, biomarkers of inflammation, and anthropometric measurements in women with obesity under a weight loss diet.

## Methods

Women with obesity aged 18 years and older who meet the study eligibility criteria were recruited from the diet therapy clinic of Abadan Imam Khomeini Hospital. Women who had a BMI outside the range of 30 to 40 kg/m^2^, were pregnant, lactating, menopausal, or suffer from food allergies, or with eating disorders, cancer, hepatic, renal, thyroid, and gastrointestinal diseases were not eligible. Women who went through considerable weight loss (> 5% of body weight) six months before the study, underwent Bariatric surgeries, or took weight management drugs, were also excluded from the study. The methodology of the study was approved by the Ethics Committee of Ahvaz University of Medical Sciences (approval number: IR.AJUMS.REC.1399.795). A signed written informed consent was obtained from each participant. The trial was registered at the Iranian Registry of Clinical Trials (www.IRCT.IR) under the registration number IRCT20201223049804N1.

**Fig. 1:**
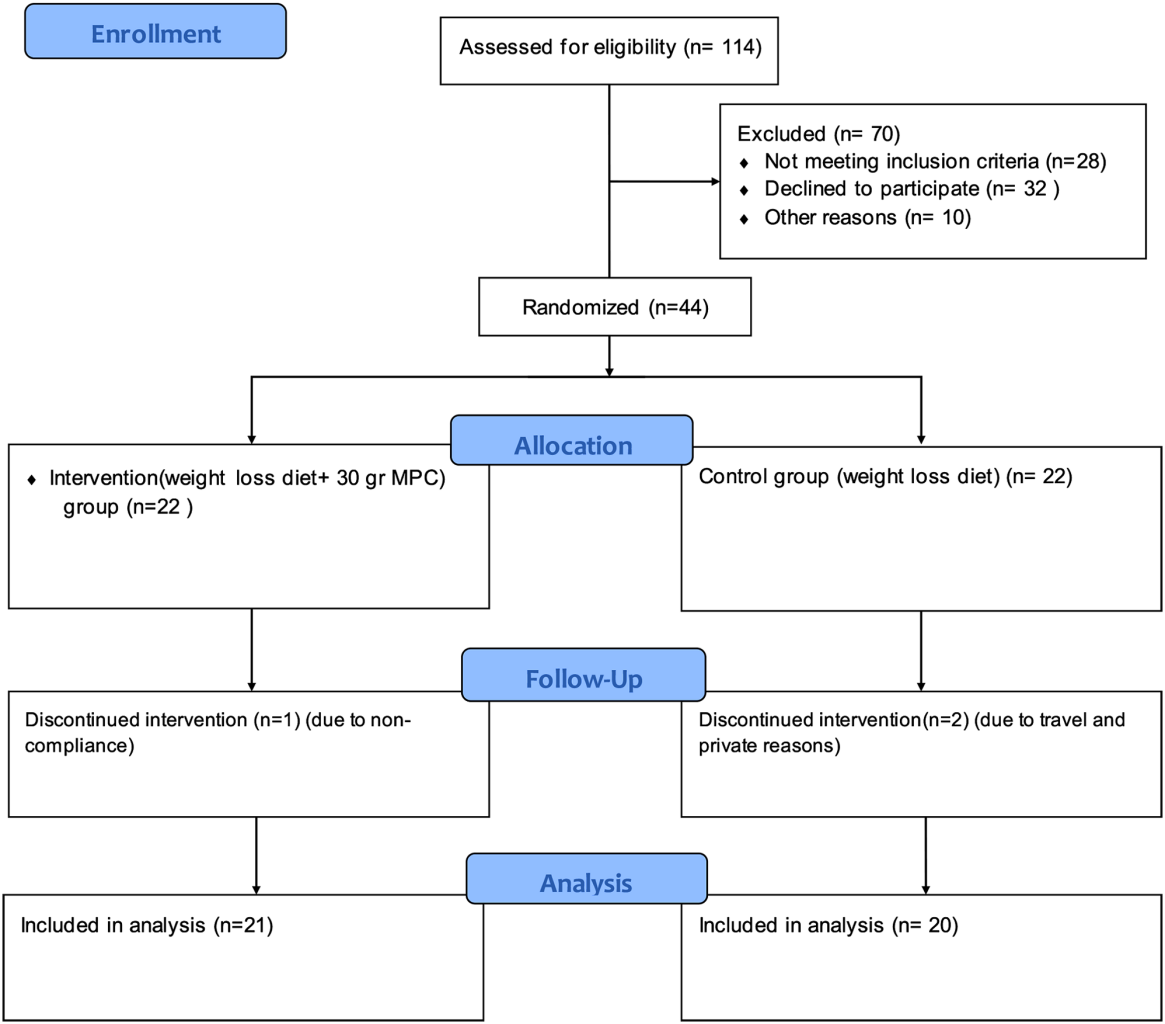
CONSORT 2010 Flow Diagram

This is a single-blind, open-labelled, parallel-group, randomized trial. The subjects were randomly stratified according to age and BMI using a permuted block randomization procedure by Random Allocation Software (RAS). The ratio of the intended number of participants in each of the matched groups was 1:1. They were assigned to one of the two study groups: (Fig. 1)

1. Standard weight loss group (*n* = 22) (control group).
2. MPC supplementation weight loss group (*n* = 22) (intervention group).

The method for sample size calculation has been published in the study protocol [21].

A dietary plan to reduce calorie intake by 800 kcal/d from the total energy expenditure (TEE) was prescribed by a trained dietitian for 8 weeks. The total energy requirements of participants were calculated using Mifflin-St. Jeor formula for basal energy expenditure (BEE), then the thermic effect of food and activity thermogenesis were added to the BEE to obtain the TEE [21]. The macronutrient distribution of the diet in the control group was as follows; 55% of energy from carbohydrates, 30% from fats, and 15% from proteins. The control group did not receive any placebo, they received only a weight-loss diet.

For the intervention group, a weight loss diet with MPC supplements was prescribed. The macronutrient distribution of the diet in the intervention group was as follows; 55% of energy from carbohydrates, 30% from fats, and 15% from proteins. Participants in the MPC group also received a 30-gram MPC powder sachet which provided 105 kcal, 0.4 g of lipid, 6 g of carbohydrate, and 20 g of protein per day. Participants were instructed to mix the sachet with 250 ml of cold water and drink it every morning on an empty stomach [21, 22]. The added calories of the MPC sachet (105 kcal) were also reduced from the TEE in the design of the weight loss intervention. Considering the calorie of each MPC sachet (105 kcal), 905 calories below estimated energy needs was regarded for the intervention group. MPC sachets were supplied by Pegah Dairy Industries Co., Khorasan, Iran. To create variety in the diet while maintaining the general principles of diet, all subjects were given a dietary exchange list and a diet according to their food habits. The study subjects were asked not to change their dietary habits and physical activity during the study. To ensure compliance, a dietitian contacted participants every week. To evaluate dietary intake, all participants completed a 3-day 24-hour dietary recall at baseline and the end of the study. Daily macro-and micro-nutrients intakes were analyzed by nutritionist IV software (First Databank, San Bruno, CA). The 8-week intervention period for this study was determined based on the Faghih et al. study which showed increasing low-fat milk consumption for 8 weeks significantly decreases general and central obesity beyond a low-calorie diet [23].

The CONSORT Flow Diagram of the RCT is shown in Fig. 1.

Anthropometric measures of participants were evaluated at baseline, week 8 of the intervention, and fortnightly during the intervention after overnight fasting with minimum clothing by the same trained dietitian. Height was measured to the nearest 0.5 cm with a tape measure in a standing position, with shoulders in a normal alignment and without shoes. Weight was measured by a digital scale without shoes and with a minimum of clothes with an accuracy of 0.1 kg. X-CONTACT 350 body composition analyzer was used to measure total body fat and fat-free mass. Participants were advised to avoid drinking water, alcohol, coffee, and tea, exercise, and bathing before the test and not to be in the menstrual phase. Waist circumference (WC) was measured at the narrowest point of the torso (precise: 0.5 cm). The body mass index (BMI) was calculated as weight (kg) divided by the square of height (m^2^). Obesity was defined as a BMI higher than 30 kg/m^2^ [24, 25]. Physical activity was evaluated using the International Physical Activity Questionnaire (IPAQ), and the results were expressed as high (> 1500 met-min/week), moderate (600–1500 met-min/week), and low (> 600 met-min/week) activity [26]. Participants were asked to maintain their usual physical activity during the investigation. Participants’ appetite was measured at baseline and the end of the study before breakfast using the Council on Nutrition Appetite Questionnaire (CNAQ). It consists of a series of scored questions evaluating appetite, hunger, feeling sick or nauseated when eating, and temperament. A total score ≥ 29 (out of 40) indicates good appetite, while a score < 28 is regarded as poor appetite [27].

Participants’ blood biomarkers were also measured at baseline and the end of the study. After 10–12 h of fasting, 10 ml of blood sample was collected from each participant. Fasting blood glucose and lipid profile were evaluated by the enzymatic method with Pars-Azmoon kits (Tehran, Iran). Insulin levels were measured by chemiluminescent immunoassay. Homeostasis model assessment-insulin resistance (HOMA-IR) was calculated by the following formula: fasting glucose (mg/dL) × fasting insulin (µU/mL) /405. ELISA kits were used to determine serum leptin and adiponectin levels (Eastbiopharm, Hangzhou, China). Criteria for glucometabolic disturbances as established by the World Health Organization (WHO) was considered: fasting blood sugar (FBS): 70–100 mg/dL: Normal; 100–125 mg/dL: Impaired fasting glucose; ≥126 mg/dL: Diabetes mellitus [28].

The data analyst was blinded after the assignment to interventions. Data were analyzed using the IBM SPSS Statistics software (Version 23) (IBM SPSS Statistics, Armonk, USA). Categorical variables were compared using the chi-square test. Continuous variables were expressed as mean ± standard deviation (SD). The Kolmogorov-Smirnov test was used to determine the normality of data distribution. For quantitative variables, the means of the two groups were compared by independent t-test and Mann-Whitney test for the parametric and nonparametric data, respectively. Paired t-test and Wilcoxon signed rank were used to compare pre- and post-intervention variables within groups. Analysis of covariance (ANCOVA) test was used to determine any differences in the MPC group at the end of the trial while adjusting for baseline values. Differences were considered significant at *p* ≤ 0.05.

## Results

One subject in the intervention group (due to noncompliance) and two participants in the control group (due to travel and private reasons) were lost to follow. All analyses were performed on 41 participants (control group, *n* = 20 and intervention group, *n* = 21). The baseline characteristics of the participants are shown in Table 1. There were no significant differences between age, weight, BMI, physical activity, and educational status of participants between the MPC and control groups (*P* > 0.05). There were also no significant differences in energy and macronutrient intakes between the two groups before and after the intervention (Table 2).

**Table 1:** Baseline characteristics of study participants

Variables	All Subject	MPC group(*n* = 21)	Control group (*n* = 20)	*P* value ^c^
**Age (years**) ^**a**^	36.95	37.19(5.77)	36.70(9.02)	0.838
**Height (cm)** ^**a**^	1.59	1.60(0.07)	1.58(0.06)	0. 408
weight	87.26	86.32(10.22)	88.20(10.32)	0.563
BMI	34.37	33.66(2.94)	35.08(3.04)	0.136
Married status				0.444
Married	25(60.97%)	14(66.66%)	11(55%)	
Unmarried	16(39.02%)	7(33.33%)	9(45%)	
**Job status** ^**b**^				0.784
Employees	10(24.39%)	6(28.57%)	4(20%)	
Non-administrative employees	9(21.95%)	4(19.04%)	5(25%)	
Housewife	22(53.65%)	11(52.38%)	11(55%)	
**Education status** ^**b**^				0.461
High school & diploma	18(43.90%)	8(38.09%)	10(50%)	
Associate degree	8(19.51%)	5(23.80%)	3(15%)	
Bachelor	13(31.70%)	6(28.57%)	7(35%)	
Postgraduate	2(4.8%)	2(9.52%)	0(0%)	
**Physical activity** ^**b**^				0.939
Low	33(90.24)	17(80.95)	16(80)	
Moderate	8(19.51)	4(19.04)	4 (20)	

a Mean (SD)

b Number (%)

c Independent t test or Mann Whitney test for numeric variables and Pearson’s chi-square test for categorical variables

**Table 2:** Daily dietary intakes of the study participants at baseline and 2 months after the intervention

Variables			MPC group (*n* = 21)	Control group (*n* = 20)	*P* value^b, c^
Energy (kcal/day)	Before		2259.80 ± 238.34 ^**a**^	2180.66 ± 279.55	0.204 ^b^
	After		1797.30 ± 142.76	1748.11 ± 139.34	0.709
	p^d^		0.001	0.001>	
Carbohydrate (% energy)	Before		51.59 ± 5.16	48.49 ± 5.93	0.062 ^b^
	After		49.92 ± 5.44	50.07 ± 6.96	0.610 ^**c**^
	p^d^		0.328	0.455	
Protein (% energy)	Before		16.47 ± 2.88	17.25 ± 3.64	0.502 ^b^
	After		18.22 ± 3.23	19.40 ± 4.37	0.811 ^**c**^
	p^d^		0.059	0.069	
Total fat (% energy)	Before		34.16 ± 5.92	36.26 ± 8.26	0.152 ^b^
	After		32.9 ± 5.57	32.35 ± 5.07	0.781 ^**c**^
	p^d^		0.492	0.110	
SFA (gr/day)	Before		14.50 ± 5.01	17.15 ± 6.53	0.146 ^b^
	After		12.09 ± 3.04	11.92 ± 4.82	0.895 ^**c**^
	p^d^		0.216	0.007	
MUFA (gr/day)	Before		17.29 ± 6.08	20.50 ± 9.85	0.208 ^b^
	After		14.62 ± 3.66	13.19 ± 3.85	0.237 ^**c**^
	p^d^		0.073	0.015	
PUFA (gr/day)	Before		24.89 ± 7.45	28.30 ± 12.47	0.283 ^b^
	After		17.65 ± 5.01	17.99 ± 5.49	0.840 ^**c**^
	p^d^		0.001	0.007	
cholesterol (gr/day)	Before		166.96 ± 122.27	193.93 ± 132.58	0.497^b^
	After		182.20 ± 122.70	188.52 ± 162.28	0.890 ^**c**^
		p^d^	0.736	0.918	

MD: mean difference, CI: confidence interval, SFA: saturated fatty acid, MUFA: monounsaturated fatty acid, PUFA: polyunsaturated fatty acid

a Mean (SD). p values of statistical significance (*p* < 0.05) are presented in bold

b Independent t test for intake of energy, carbohydrate, protein, and total fat and Mann-Whitney U for SFA, MUFA, PUFA, and other fat

c Analysis of covariance (adjusted for baseline values and changes in intake of energy, percent of carbohydrate, protein, total fat, SFA, MUFA, PUFA, and other fat )

d Paired t test intake of energy, carbohydrate, protein, and total fat and Wilcoxon for SFA, MUFA, PUFA, and other fat

After eight weeks of supplementation with 30 g of MPC, participants in the intervention group had significantly lower BMI (*P* = 0.009), WC (*P* = 0.013), and fat mass (*P* = 0.021) compared to the control group. However, the differences in weight (*P* = 0.137) and fat-free mass (*P* = 0.818) did not reach a statistically significant level. Within-group differences also suggested significant reductions in body weight, BMI, WC, and fat mass after intervention in both groups but changes in fat-free mass did not reach a statistically significant level in the MPC group (*P* = 0.082) (Table 3).

**Table 3:** Anthropometric measures at baseline and end of the intervention

Variables		MPC group (*n* = 21)	Control group (*n* = 20)	*P* value^b, c^
Body weight (kg)	Before	86.12 ± 10.22 ^**a**^	88.20 ± 10.32	0.563^**b**^
	After	81.61 ± 9.17	85.59 ± 10.27	0.137 ^**c**^
	p^d^	< 0.001	< 0.001	
BMI (kg/m^2^)	Before	33.66 ± 2.94	35.08 ± 3.05	0.136 ^**b**^
	After	31.57 ± 2.39	34.05 ± 3.21	0.009 ^**c**^
	p^d^	< 0.001	< 0.001	
WC (cm)	Before	98.40 ± 4.24	100.42 ± 7.99	0.315 ^**b**^
	After	93.35 ± 3.47	98.15 ± 7.66	0.013 ^**c**^
	p^d^	< 0.001	< 0.001	
Body fat (kg)	Before	35.45 ± 5.66	36.76 ± 5.34	0.452 ^**b**^
	After	30.92 ± 5.72	35.20 ± 5.52	0.021 ^**c**^
	p^d^	< 0.001	0.002	
Body fat-free mass (%)	Before	50.86 ± 5.12	51.44 ± 5.57	0.731 ^**b**^
	After	50.15 ± 4.54	50.39 ± 5.48	0.818 ^**c**^
	p^d^	0.082	< 0.001	

MD: mean difference, CI: confidence interval, BMI: body mass index, WC: waist circumference

aMean (SD). p values of statistical significance (*p* < 0.05) are presented in bold

bIndependent t test

cAnalysis of covariance (adjusted for baseline values and changes in intake of energy, percent of carbohydrate, protein, total fat, SFA, MUFA, PUFA, and other fat, physical activity, and baseline values)

dPaired t test

Participants in the MPC group also had a lower FBS (*P* < 0.001), insulin (*P* = 0.027), HOMA-IR (*P* = 0.020), LDL-C (*P* = 0.025), leptin (*P* = 0.014), appetite score (*P* = 0.002), and higher adiponectin (*P* = 0.032), and HDL-C (*P* = 0.001) serum levels following the intervention compared to the control group (Table 4). Appetite and biochemical markers were all reduced significantly following MPC supplementation, but the changes were not significant in the control group (except for a reduction in insulin, total cholesterol, and LDL-C and an increase in adiponectin) (*P* > 0.05).

**Table 4:** Appetite and biochemical markers at baseline and at the end of the intervention

Variables		MPC group(*n* = 21)	Control group (*n* = 20)	*P* value^b, c^
Leptin	Before	97.05 ± 14.77 ^**a**^	97.55 ± 18.18	0.923 ^**b**^
	After	81.68 ± 12.93	94.02 ± 16.73	0.014 ^**c**^
	p^d^	< 0.001	0.052	
Adiponectin	Before	13.32 ± 3.42	12.45 ± 3.27	0.415 ^**b**^
	After	16.66 ± 4.49	13.77 ± 3.77	0.032 ^**c**^
	p^d^	0.001	0.007	
FBS (mg/dl)	Before	96.05 ± 10.24	99.80 ± 15.11	0.365 ^**b**^
	After	87.62 ± 8.56	97.90 ± 10.56	< 0.001 ^**c**^
	p^d^	< 0.001	0.352	
Insulin (µIU/ml)	Before	11.03 ± 4.08	11.51 ± 3.56	0.688 ^**b**^
	After	7.63 ± 3.69	9.88 ± 2.60	0.027 ^**c**^
	p^d^	< 0.001	0.038	
HOMA-IR	Before	2.43 ± 1.05	2.81 ± 1.01	0.241 ^**b**^
	After	1.81 ± 0.86	2.47 ± 0.088	0.020 ^**c**^
	p^d^	0.002	0.089	
TC (mg/dl)	Before	185.84 ± 27.94	188.98 ± 9.35	0.635 ^**b**^
	After	167.98 ± 13.18	174.06 ± 17.63	0.187 ^**c**^
	p^d^	0.012	< 0.001	
LDL-C (mg/dl)	Before	112.38 ± 28.96	114.07 ± 15.09	0.818 ^**b**^
	After	91.94 ± 11.90	102.70 ± 17.94	0.025 ^**c**^
	p^d^	< 0.001	0.017	
HDL-C (mg/dl)	Before	39.42 ± 6.50	38.95 ± 6.25	0.812 ^**b**^
	After	45.90 ± 5.23	39.65 ± 6.24	0.001 ^**c**^
	p^d^	0.001	0.661	
TG(mg/dl)	Before	170.12 ± 70.17	179.81 ± 72.47	0.666 ^**b**^
	After	150.63 ± 50.98	158.56 ± 65.52	0.707 ^**c**^
	p^d^	0.304	0.153	
Appetite	Before	28.62 ± 4.73	28.80 ± 4.08	0.896 ^**b**^
	After	23.66 ± 2.90	27.05 ± 3.53	0.002 ^**c**^
	p^d^	> 0.001	0.098	

MD: mean difference, CI: confidence interval, TC: total cholesterol, LDL-C: low-density lipoprotein cholesterol,

HDL-C high-density lipoprotein cholesterol, TG triglycerides, FBS fasting blood sugar, HOMA-IR homeostasis model assessment for insulin resistance

a Mean (SD). p values of statistical significance (*p* < 0.05) are presented in bold

b Independent t test for TC, HDL-c, LDL-c, TG, and FBS and Mann-Whitney U for insulin, and HOMA-IR

c Analysis of covariance (adjusted for baseline values and changes in intake of energy, percent of carbohydrate, protein, total fat, SFA, MUFA, PUFA, and other fat and physical activity, and baseline values)

d Paired t test for TC, HDL-c, LDL-c, TG, and FBS and Wilcoxon for insulin, and HOMA-IR

## Discussion

The findings of this study suggest that supplementation with 30 g MPC per day for 8 weeks in women with obesity following a weight-loss diet resulted in reductions in BMI, WC, fat mass, FBS, insulin, LDL-C, and leptin, and an increase in HDL-C and adiponectin. The recommended dietary allowance (RDA) for protein to prevent deficiency for an average sedentary adult is 0.8 g per kilogram of body weight [29]. In the present study, protein intake was almost at the recommended amount (0.8 g/kg to 0.89 g/kg). A diet high in protein may help improve anthropometric measures and metabolic markers in overweight and obese individuals [30] by increasing satiety and dietary-induced thermogenesis (DIT) [31]. However, the beneficial effects observed may depend on the source of dietary protein [32]. For example, whey protein has been shown to have a higher thermic effect compared to soy protein. This could be due to the higher content of branched-chain amino acids found in whey protein [33]. While satiety and appetite are not interchangeable, the lower appetite score observed following MPC supplementation in this study supports the high post-prandial satiety reported for whey protein compared to fish and eggs previously [34]. An increase in serum level of cholecystokinin following the consumption of whey protein [35] can also induce satiety. Casein, another major contributor to milk protein also induces long-term satiety [36]. Moreover, the favorable impact of MPC on appetite sensations during weight loss may be related to the changes in blood leptin, as reflected by the between-group differences in its circulating concentrations. Leptin is a key regulator of appetite, food intake, and body weight. Leptin is also an important factor in energy homeostasis, metabolism and adiposity [37]. An increase in protein intake enhances the CNS leptin sensitivity and results in clinically significant weight loss [38].

The lipid profile, body fat, and WC improvements reported in this study could also be justified by the reduction of leptin and the increase in serum adiponectin levels observed following MPC supplementation. Similar findings were reported following a high-dairy diet (three servings per day) on fat and WC in obese African-American adults [39]. Weight and fat loss on the high dairy diet were 2-fold higher, and loss of lean body mass was markedly reduced compared to the low dairy diet (one serving per day). In another study, semi-skimmed milk intake (1 L/day) for six months significantly reduced visceral adipose tissue and liver fat compared to a soft drink with a similar energy intake [40]. Josse et al. aimed to determine how daily exercise (resistance and/or aerobic) and a hypo-energy diet varying in protein and calcium content from dairy foods would affect the composition of weight lost in otherwise healthy, premenopausal, overweight, and obese women. Ninety participants were randomized to 3 groups (*n* = 30/group): high protein, high dairy, adequate protein, medium dairy, and adequate protein, low dairy differing in the quantity of total dietary protein and dairy food-source protein consumed: 30 and 15%, 15 and 7.5%, or 15 and < 2% of energy, respectively. A weight loss diet with higher protein and increased dairy intake compared to one with lower protein and dairy led to more favorable body composition changes in women. Similarly, milk intake with enhanced protein content resulted in reduced blood glucose and increased postprandial satiety more significantly than regular milk [41]. In contrast, A large meta-analysis of 27 clinical studies reported that dairy consumption had no impact on weight change in the long term [42]. However, in some of the studies included in this meta-analysis, the participants did not receive any diet counselling, and their energy intake was not restricted. In some cases, they even had a greater energy intake than the control group [43, 44]. Overall, it appears that supplementation with milk protein together with a weight loss diet is effective in improving lipid profile, body fat, and WC. As mentioned above, Whey protein has higher amounts of branched chained amino acids leucine and isoleucine, glycine, lysine, and cysteine. The anti-inflammatory properties of glycine have been shown in some studies; specifically reduced in Interleukin-6 and Tumour necrosis factor α gene expression in addition to elevation in adiponectin and Interleukin-10 gene expression in monocytes and adipose tissue [45–47]. Moreover, the production of adiponectin in adipocyte cells can be modulated by leucine, as demonstrated [48]. Overall, the distinctive amino acid composition of dairy products can regulate the production and gene expression of cytokines; but the use of dairy amino acids in studies on inflammatory biomarkers is limited.

The findings of this study, however, did not show any significant effect of MPC supplementation on fat-free mass. Literature suggests that animal proteins, especially dairy proteins, could support muscle protein synthesis more than plant proteins in the long term [49]. In a meta-analysis of 23 clinical trials, of which 20 investigated the effects of milk proteins, it was shown that protein supplementation positively impacted muscle mass [50]. While similar effects were not observed in the current study, it should be noted that participants in the current study followed a weight loss diet and were asked to maintain their physical activity and exercise level. Weight loss generally accompanies the loss of muscle mass [51]. Physical activity and/or exercise are also needed for proteins to exert their anabolic effects [52, 53].

The beneficial effect of MPC on reducing FBS and insulin levels observed in this study also aligns with previous studies. In a recent study, four servings per day of low-fat milk and yogurt reduced fasting plasma insulin concentrations and improved insulin resistance in healthy adults [54]. In a diet-induced obese rat model, whole milk supplementation resulted in a better glycaemic control and lipid profile than whey or casein supplementation separately [18]. Another study reported that intraduodenal infusion of MPC significantly improved the effects of sitagliptin including glycemic and short-term food intake suppression. The results of this study confirm the hypothesis that the consumption of dairy protein may be useful as a complementary therapy to enhance the glycemic and food intake suppressive effects of GLP-1-based pharmacotherapies [55]. The intraintestinal presence of specific bioactive components, whole proteins, and select amino acids found within MPC is linked with insulin and gut peptide secretions, as well as suppression of food intake [35, 56, 57]. However, in another study with a lower dose of MPC supplementation (14 g per day), significant changes in blood lipids or insulin resistance were not reported [58]. This could indicate that MPC’s beneficial effects may be dose-dependent.

To the best of our knowledge, the present study was the first to examine the effects of MPC on a wide range of anthropometric and metabolic markers in women with obesity under a weight loss diet. However, the study had some limitations. As mentioned in the methodology, dietary intake in this investigation was assessed through a 3-day dietary recall. It is well-established that underreporting and recall bias is a prevalent phenomenon, particularly among individuals who are overweight or obese. Hence, it is recommended that future studies employ three-day dietary records to enhance the precision of their findings. Only women were included in this study which limits the generalisation of findings. To distinguish the specific effects of the MPC intake versus the general increase in protein consumption on weight loss outcomes, we recommend adding a study group with the same protein amount as in the MPC group but without MPC additive in future studies. Also to determine if there are any delayed or cumulative effects of dairy consumption on weight change over a longer duration, it is recommended that future studies extend the duration of intervention.

Also, investigating the serum levels of other satiety-regulating hormones, such as glucagon-like peptide-1 (GLP-1) could clarify the MPC effects on appetite and satiety observed. Analysis of dose-dependent effects was not possible due to the limited number of participants. Therefore, it is not possible to identify the effective dose of MPC supplementation. Also, it is important to note that high protein diets may not suit everyone, especially in people with chronic kidney disease due to the extra load caused by the removal of protein metabolism by-products (urea). Therefore, high protein diet/supplementation should be taken under medical advice from health professionals.

## Conclusions

In conclusion, this study indicates that supplementation with 30 g of MPC daily for 8 weeks could significantly improve some anthropometric and metabolic markers and hormones in dieting women with obesity. This could be due to the satiety effects and thermogenesis caused by milk proteins and their specific amino acid content. In addition, the results of this trial can help women with obesity to reduce weight and improve their cardiometabolic health. However, larger controlled trials investigating the effect of different doses of MPC in both genders, analysis of dose-dependent effects, and investigation of the serum levels of other satiety-regulating hormones is required to clarify the findings of this study.

## Data Availability

The data that support the findings of this study are available from the corresponding author upon reasonable request.

## References

[1] Ryan D, Barquera S, Barata Cavalcanti O, Ralston J (2021). The global pandemic of overweight and obesity: addressing a twenty-first century multifactorial disease.

[2] Jafari-Adli S, Jouyandeh Z, Qorbani M, Soroush A, Larijani B, Hasani-Ranjbar S (2014). Prevalence of obesity and overweight in adults and children in Iran; a systematic review. J Diabetes Metabolic Disorders.

[3] Gustafson B, Hammarstedt A, Andersson CX, Smith U, Arteriosclerosis (2007). Thromb Vascular Biology.

[4] Ojo O. Nutrition and chronic conditions. MDPI; 2019. p. 459. 10.3390/nu11020459 PMC6412662 30813286

[5] Westerterp KR (2004). Diet induced thermogenesis. Nutr Metabolism.

[6] Veldhorst M, Smeets A, Soenen S, Hochstenbach-Waelen A, Hursel R, Diepvens K (2008). Protein-induced satiety: effects and mechanisms of different proteins. Physiol Behav.

[7] Westerterp-Plantenga M, Luscombe-Marsh N, Lejeune M, Diepvens K, Nieuwenhuizen A, Engelen M (2006). Dietary protein, metabolism, and body-weight regulation: dose–response effects. Int J Obes.

[8] Gilbert J-A, Bendsen N, Tremblay A, Astrup A (2011). Effect of proteins from different sources on body composition. Nutr Metabolism Cardiovasc Dis.

[9] Mathai JK, Liu Y, Stein HH (2017). Values for digestible indispensable amino acid scores (DIAAS) for some dairy and plant proteins may better describe protein quality than values calculated using the concept for protein digestibility-corrected amino acid scores (PDCAAS). Br J Nutr.

[10] Crichton G, Bryan J, Buckley J, Murphy K (2011). Dairy consumption and metabolic syndrome: a systematic review of findings and methodological issues. Obes Rev.

[11] Rice BH, Cifelli CJ, Pikosky MA, Miller GD (2011). Dairy components and risk factors for cardiometabolic syndrome: recent evidence and opportunities for future research. Adv Nutr.

[12] Pereira MA, Jacobs DR, Van Horn L, Slattery ML, Kartashov AI, Ludwig DS (2002). Dairy consumption, obesity, and the insulin resistance syndrome in young adults: the CARDIA Study. JAMA.

[13] Haidari F, Aghamohammadi V, Mohammadshahi M, Ahmadi-Angali K (2017). Effect of whey protein supplementation on levels of endocannabinoids and some of metabolic risk factors in obese women on a weight-loss diet: a study protocol for a randomized controlled trial. Nutr J.

[14] Haidari F, Aghamohammadi V, Mohammadshahi M, Ahmadi-Angali K, Asghari-Jafarabadi M (2020). Whey protein supplementation reducing fasting levels of anandamide and 2-AG without weight loss in pre-menopausal women with obesity on a weight-loss diet. Trials.

[15] Tong X, Dong JY, Wu ZW, Li W, Qin LQ (2011). Dairy consumption and risk of type 2 diabetes mellitus: a meta-analysis of cohort studies. Eur J Clin Nutr.

[16] Zhang J, Liu D, Liu Y, Yu Y, Hemar Y, Regenstein JM (2020). Effects of particle size and aging of milk protein concentrate on the biophysical properties of an intermediate-moisture model food system. Food Bioscience.

[17] Nilsson M, Stenberg M, Frid AH, Holst JJ, Björck IM (2004). Glycemia and insulinemia in healthy subjects after lactose-equivalent meals of milk and other food proteins: the role of plasma amino acids and incretins. Am J Clin Nutr.

[18] Eller LK, Reimer RA (2010). Dairy protein attenuates weight gain in obese rats better than whey or casein alone. Obesity.

[19] Norris GH, Milard M, Michalski M-C, Blesso CN (2019). Protective properties of milk sphingomyelin against dysfunctional lipid metabolism, gut dysbiosis, and inflammation. J Nutr Biochem.

[20] Ostadrahimi A, Taghizadeh A, Mobasseri M, Farrin N, Payahoo L, Gheshlaghi ZB (2015). Effect of probiotic fermented milk (kefir) on glycemic control and lipid profile in type 2 diabetic patients: a randomized double-blind placebo-controlled clinical trial. Iran J Public Health.

[21] Haidari F, Elahikhah M, Islam SMS, Mohammadshahi M, Shahbazian H, Aghamohammadi V (2022). Effects of milk protein concentrate supplementation on metabolic parameters, adipocytokines and body composition in obese women under weight-loss diet: study protocol for a randomised controlled trial. BMJ open.

[22] Haidari F, Aghamohammadi V, Mohammadshahi M, Ahmadi-Angali K, Asghari-Jafarabadi M (2020). Whey protein supplementation reducing fasting levels of anandamide and 2-AG without weight loss in pre-menopausal women with obesity on a weight-loss diet. Trials.

[23] Faghih S, Abadi A, Hedayati M, Kimiagar S (2011). Comparison of the effects of cows’ milk, fortified soy milk, and calcium supplement on weight and fat loss in premenopausal overweight and obese women. Nutr Metabolism Cardiovasc Dis.

[24] Sikaris KA (2004). The clinical biochemistry of obesity. Clin Biochemist Reviews.

[25] Pourghassem Gargari B, Aliasgharzadeh A (2011). Effect of folic acid supplementation on indices of glycemic control, insulin resistance and lipid profile in patients with type 2 diabetes mellitus. Iran J Endocrinol Metabolism.

[26] Aadahl M, Jørgensen T (2003). Validation of a new self-report instrument for measuring physical activity. Med Sci Sports Exerc.

[27] Wilson M-MG, Thomas DR, Rubenstein LZ, Chibnall JT, Anderson S, Baxi A (2005). Appetite assessment: simple appetite questionnaire predicts weight loss in community-dwelling adults and nursing home residents. Am J Clin Nutr.

[28] Ryden L, Standl E, Bartnik M, Van den Berghe G, Betteridge J, De Boer M-J (2007). Guidelines on diabetes, pre-diabetes, and cardiovascular diseases: executive summary: the Task Force on Diabetes and Cardiovascular diseases of the European Society of Cardiology (ESC) and of the European Association for the Study of Diabetes (EASD). Eur Heart J.

[29] Wu G (2016). Dietary protein intake and human health. Food Funct.

[30] Leidy HJ, Clifton PM, Astrup A, Wycherley TP, Westerterp-Plantenga MS, Luscombe-Marsh ND (2015). The role of protein in weight loss and maintenance. Am J Clin Nutr.

[31] Moon J, Koh G (2020). Clinical evidence and mechanisms of high-protein Diet-Induced weight loss. J Obes Metab Syndr.

[32] Larsen TM, Dalskov S-M, van Baak M, Jebb SA, Papadaki A, Pfeiffer AF (2010). Diets with high or low protein content and glycemic index for weight-loss maintenance. N Engl J Med.

[33] Acheson KJ, Blondel-Lubrano A, Oguey-Araymon S, Beaumont M, Emady-Azar S, Ammon-Zufferey C (2011). Protein choices targeting thermogenesis and metabolism. Am J Clin Nutr.

[34] Pal S, Ellis V (2010). The acute effects of four protein meals on insulin, glucose, appetite and energy intake in lean men. Br J Nutr.

[35] Hall W, Millward D, Long S, Morgan L (2003). Casein and whey exert different effects on plasma amino acid profiles, gastrointestinal hormone secretion and appetite. Br J Nutr.

[36] Alfenas RCG, Bressan J, Paiva AC (2010). Effects of protein quality on appetite and energy metabolism in normal weight subjects. Arquivos Brasileiros De Endocrinologia Metabologia.

[37] Obradovic M, Sudar-Milovanovic E, Soskic S, Essack M, Arya S, Stewart AJ (2021). Leptin and obesity: role and clinical implication. Front Endocrinol.

[38] Weigle DS, Breen PA, Matthys CC, Callahan HS, Meeuws KE, Burden VR (2005). A high-protein diet induces sustained reductions in appetite, ad libitum caloric intake, and body weight despite compensatory changes in diurnal plasma leptin and ghrelin concentrations–. Am J Clin Nutr.

[39] Zemel MB, Richards J, Milstead A, Campbell P (2005). Effects of calcium and dairy on body composition and weight loss in African-American adults. Obes Res.

[40] Maersk M, Belza A, Stødkilde-Jørgensen H, Ringgaard S, Chabanova E, Thomsen H (2012). Sucrose-sweetened beverages increase fat storage in the liver, muscle, and visceral fat depot: a 6-mo randomized intervention study. Am J Clin Nutr.

[41] Josse AR, Atkinson SA, Tarnopolsky MA, Phillips SM (2011). Increased Consumption of Dairy Foods and protein during Diet- and Exercise-Induced weight loss promotes Fat Mass loss and lean Mass Gain in overweight and obese Premenopausal women. J Nutr.

[42] Chen M, Pan A, Malik VS, Hu FB (2012). Effects of dairy intake on body weight and fat: a meta-analysis of randomized controlled trials. Am J Clin Nutr.

[43] Barr SI, McCARRON DA, Heaney RP, Dawson-Hughes B, Berga SL, Stern JS (2000). Effects of increased consumption of fluid milk on energy and nutrient intake, body weight, and cardiovascular risk factors in healthy older adults. J Am Diet Assoc.

[44] Wennersberg MH, Smedman A, Turpeinen AM, Retterstøl K, Tengblad S, Lipre E (2009). Dairy products and metabolic effects in overweight men and women: results from a 6-mo intervention study. Am J Clin Nutr.

[45] Garcia-Macedo R, Sanchez-Muñoz F, Almanza-Perez JC, Duran-Reyes G, Alarcon-Aguilar F, Cruz M (2008). Glycine increases mRNA adiponectin and diminishes pro-inflammatory adipokines expression in 3T3-L1 cells. Eur J Pharmacol.

[46] Spittler A, Reissner CM, Oehler R, Gornikiewicz A, Gruenberger T, Manhart N (1999). Immunomodulatory effects of glycine on LPS-treated monocytes: reduced TNF‐α production and accelerated IL‐10 expression. FASEB J.

[47] Da Silva MS, Rudkowska I (2015). Dairy nutrients and their effect on inflammatory profile in molecular studies. Mol Nutr Food Res.

[48] Sun X, Zemel MB (2007). Leucine and calcium regulate fat metabolism and energy partitioning in murine adipocytes and muscle cells. Lipids.

[49] Gilbert JA, Bendsen NT, Tremblay A, Astrup A (2011). Effect of proteins from different sources on body composition. Nutr Metab Cardiovasc Dis.

[50] Cermak NM, Res PT, de Groot LC, Saris WHM, van Loon LJC (2012). Protein supplementation augments the adaptive response of skeletal muscle to resistance-type exercise training: a meta-analysis. Am J Clin Nutr.

[51] Willoughby D, Hewlings S, Kalman D (2018). Body composition changes in weight loss: strategies and supplementation for maintaining lean body mass, a brief review. Nutrients.

[52] Cermak NM, de Groot LC, Saris WH, Van Loon LJ (2012). Protein supplementation augments the adaptive response of skeletal muscle to resistance-type exercise training: a meta-analysis. Am J Clin Nutr.

[53] Hidayat K, Chen G-C, Wang Y, Zhang Z, Dai X, Szeto I (2018). Effects of milk proteins supplementation in older adults undergoing resistance training: a meta-analysis of randomized control trials. J Nutr Health Aging.

[54] Rideout TC, Marinangeli CP, Martin H, Browne RW, Rempel CB (2013). Consumption of low-fat dairy foods for 6 months improves insulin resistance without adversely affecting lipids or bodyweight in healthy adults: a randomized free-living cross-over study. Nutr J.

[55] Olivos DR, McGrath LE, Turner CA, Montaubin O, Mietlicki-Baase EG, Hayes MR (2014). Intraduodenal milk protein concentrate augments the glycemic and food intake suppressive effects of DPP-IV inhibition. Am J Physiology-Regulatory Integr Comp Physiol.

[56] Diepvens K, Häberer D, Westerterp-Plantenga M (2008). Different proteins and biopeptides differently affect satiety and anorexigenic/orexigenic hormones in healthy humans. Int J Obes.

[57] Liddle RA, Green GM, Conrad CK, Williams JA (1986). Proteins but not amino acids, carbohydrates, or fats stimulate cholecystokinin secretion in the rat. Am J Physiology-Gastrointestinal Liver Physiol.

[58] Bendtsen LQ, Lorenzen JK, Larsen TM, van Baak M, Papadaki A, Martinez JA (2014). Associations between dairy protein intake and body weight and risk markers of diabetes and CVD during weight maintenance. Br J Nutr.

